# Experience in laparoscopic transcystic common bile duct exploration for super-elderly patients with choledocholithiasis—A 96-year-old case report

**DOI:** 10.1016/j.heliyon.2024.e41204

**Published:** 2024-12-12

**Authors:** Zongming Zhang, Limin Liu, Chong Zhang, Zhuo Liu, Yue Zhao, Hui Qi, Haiyan Yang, Baijiang Wan, Mingwen Zhu, Hai Deng, Jinqiu Feng, Fucheng Liu, Zhentian Guo, Peijie Yao

**Affiliations:** a Department of General Surgery, Beijing Electric Power Hospital, State Grid Corporation of China, Capital Medical University, Beijing, 100073, China; b Key Laboratory of Geriatrics (Hepatobiliary Diseases), China General Technology Group, Beijing, 100073, China

**Keywords:** ACC (acute calculous cholecystitis), LTCBDE (laparoscopic transcystic common bile duct exploration), Super-elderly patients, CBD (common bile duct), CBDS (common bile duct stone)

## Abstract

**Background:**

Super-elderly patients with choledocholithiasis are considered to be at high risk for undergoing surgery. While laparoscopic transcystic common bile duct exploration (LTCBDE) is regarded as a challenging procedure for super-elderly patients with choledocholithiasis, there have been no reported cases of its use in super-elderly patients over the age of 96.

**Case summary:**

This case study presents the case of a 96-year-old female patient with acute calculous cholecystitis and choledocholithiasis. Despite the presence of multiple high-risk comorbidities, the patient underwent with laparoscopic cholecystectomy (LC) plus LTCBDE, with appropriate perioperative safety measures in place, and made a full recovery, being discharged from the hospital on the seventh day following the operation.

**Conclusion:**

The case study demonstrates the successful treatment of a 96-year-old patient with choledocholithiasis via LTCBDE, utilising skilled laparoscopic and choledochoscopic techniques along with robust perioperative safety measures. This achievement sets a historical precedent for the successful treatment of a 96-year-old patient with choledocholithiasis via LTCBDE, both domestically and internationally.

## 1 Introduction

The principal trend in global population growth is an aging population. While a number of developed countries experienced an earlier transition to an aging society, developing countries such as China are currently experiencing the fastest growth rate and the largest overall number of elderly people. The phenomenon of population aging has had a profound impact on all generations within society.

Cholelithiasis is currently one of the most common diseases and occurs in 10–20 % of the world's population. Choledocholithiasis remains a difficult task to date. One of the most important problems of biliary tract surgery is the treatment of choledocholithiasis, the frequency of which in cholelithiasis varies from 10 to 38 % [1]. Laparoscopic common bile duct exploration (LCBDE) has been becoming more and more popular in patients with symptomatic choledocholithiasis. However, the safety and efectiveness of LCBDE in elderly patients with choledocholithiasis is still uncertain [2]. In recent years, laparoscopic transcystic common bile duct (CBD) exploration (LTCBDE) has emerged as a promising minimally invasive treatment for cholecystolithiasis complicated with choledocholithiasis [3]. However, the success rate varies considerably between domestic and international reports, and the surgical risks for elderly patients, particularly those over 90 years old, are often challenging [[4], [5], [6]]. To date, there have been no reported cases of LTCBDE treatment for choledocholithiasis in super-elderly patients over the age of 96.

Just recently, we successfully treated a 96-year-old female with acute calculous cholecystitis combined with common bile duct stones (CBDS) by means of LTCBDE and comprehensive perioperative safety assurance. Our review of the scientific and technological literature revealed that this case set a historical precedent for the highest age of 96 years for the successful treatment of super-elderly patients with choledocholithiasis by LTCBDE both domestically and internationally [7]. This case is hereby reported in detail.

## 2 Case report

### 2.1 Chief complaints

A 96-year-old female patient, born on February 22, 1928, with a 14-day history of right upper abdominal pain and fever was admitted to our hospital on May 7, 2024.

### 2.2 History of present illness

The patient presented with sudden postprandial right upper abdominal pain and fever (maximum temperature 38.6 °C) 14 days ago, subsequently requiring admission to a medical facility in Beijing on April 23, 2024. An abdominal CT revealed the presence of gallbladder stones and CBDS. Her peripheral blood leukocyte count (WBC) was 14.2 × 10^9^/L, with a neutrophil percentage of 90.70 %, and high-sensitive C-reactive protein (Hs-CRP) at 145.33 mg/L. Diagnosed with acute cholangitis, CBDS, and gallbladder stones, after administration of anti-infective, intravenous infusion, and symptomatic treatments, the patient improved and was discharged from the hospital 7 days later, on April 30.

Following discharge, the patient continues to experience recurrent abdominal discomfort in the upper right quadrant. In order to pursue a cure, she sought treatment at our hospital, drawn by admiration for our facility.

### 2.3 History of past illness

She had a history of hypertension, coronary heart disease, and hyperlipidemia for 20 years; cataracts and glaucoma in her right eye for 10 years, blindness for 6 years; and deep vein thrombosis in the lower limbs and the pulmonary embolism for 10 months. She had been taking Rivaroxaban long-term until the day of admission.

### 2.4 Personal and family history

No history of smoking, drinking, or family disease.

### 2.5 Physical examination

Her abdomen was soft with tenderness in the right upper quadrant, no rebound pain, and a positive Murphy's sign. Ascites sign was negative. Her blood pressure was 138/87 mmHg, pulse 116 beats/min and respiratory rate 30 breaths/min.

### 2.6 Laboratory examinations

Blood gas analysis, cardiac function, liver function, blood routine, coagulation function, high-sensitive C-reactive protein (Hs-CRP), and procalcitonin (PCT) were checked at 6 a.m. on the day of surgery, with the results in Table 1.

**Table 1:** Laboratory examinations of the patient.

Items	Results	Units
Blood gas analysis		
partial pressure of oxygen (PO_2_)	84.7	mmHg
oxygen saturation (SO_2_)	96.1 %	
partial pressure of carbon dioxide (PCO_2_)	31.9	mmHg
Cardiac function		
high-sensitive troponin-I (HSTnI)	0.011	ng/mL
creatine kinase isoenzyme (CKMB)	0.5	ng/mL
myoglobin (Myo)	44.9	ng/mL
B-type natriuretic peptide (BNP)	51.6	pg/mL
Liver function		
alanine aminotransferase (ALT)	7	U/L
albumin (Alb)	40.1	g/L
total bilirubin (Tbil)	12.37	μmol/L
direct bilirubin (DBil)	2.24	μmol/L
Blood routine		
peripheral blood leukocyte count (WBC)	6.4 × 10^9^	/L
neutrophil percentage (N%)	75.1 %	
Coagulation function		
prothrombin time (PT)	18.5	s
international normalized ratio (INR)	1.71	
D-dimer	0.86	mg/L
High-sensitive C-reactive protein (Hs-CRP)	41.28	mg/L
Procalcitonin (PCT)	0.08	ng/mL

### 2.7 Imaging examinations

Chest CT: Bilateral bronchitis, inflammatory lesions in the lower lobe of the left lung.

**Fig. 1:**
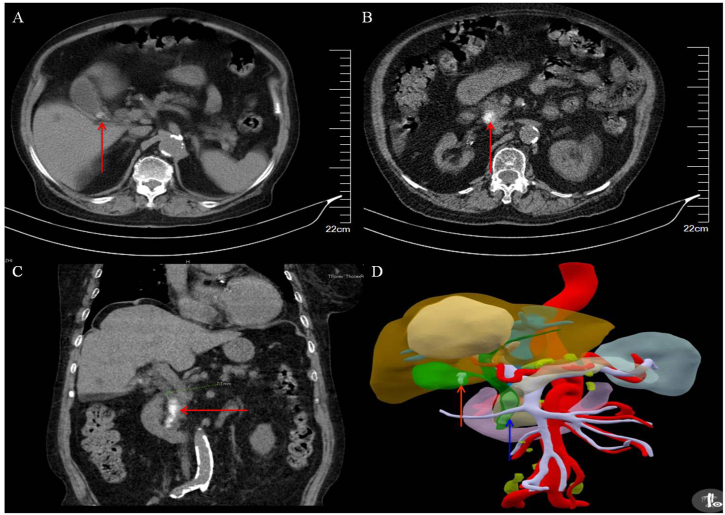
Preoperative CT in a 96-year-old female patient with ACC + CBDS. A: Preoperative axial CT image showed a circular (diameter 8 mm, arrow) high-density shadow at the neck of gallbladder, with a bilateral sign on the gallbladder wall; B: a circular (diameter 15 mm, arrow) high-density shadow within the CBD; C: Coronal CT image displayed multiple nodular high-density shadows in the CBD; D: CT three-dimensional (3D) visualization reconstruction image confirmed the smaller high-density shadow at the neck of the gallbladder (red arrow), and the larger high-density shadows in the CBD (blue arrow). ACC: Acute calculous cholecystitis. CT: Computed tomography. CBD: Common bile duct. CBDS: Common bile duct stone.

Abdominal CT: Axial image showed a circular (diameter 8 mm) high-density shadow at the neck of the gallbladder, with a bilateral sign on the gallbladder wall (Fig. 1A); A circular (diameter 15 mm) high-density shadow within the CBD (Fig. 1B); Coronal image showed multiple nodular high-density shadows in the CBD (Fig. 1C); Three-dimensional (3D) visualization reconstruction image confirmed the smaller high-density shadow at the neck of the gallbladder and the larger high-density shadows in the CBD (Fig. 1D).

### 2.8 Treatment

Following an active preoperative cardiopulmonary function evaluation, the patient underwent laparoscopic cholecystectomy (LC) plus and laparoscopic transcystic CBD exploration (LTCBDE) on May 13, 2024, with no surgical contraindications.

**Fig. 2:**
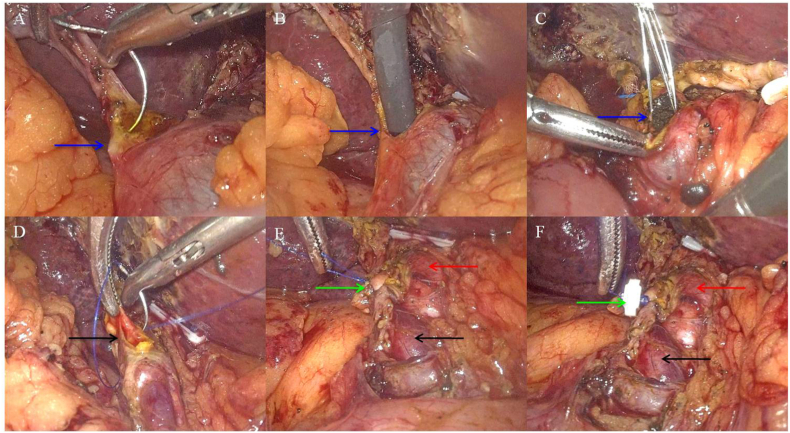
Intraoperative images of LC + LTCBDE in the 96-year-old female patient with ACC + CBDS. A: The cystic duct is relatively thin (diameter 5 mm), and the suture needle puncture incision method is used to cut the cystic duct (arrow); B: A choledochoscope (Olympus CHF type 5, Japan) was inserted into the CBD via the cystic duct after dilating the Heister valve (arrow); C: The crushed CBDS (by means of applying pressure and water flushing, pushing and crushing) were removed through the choledochoscopic stone removal basket (arrow); D: Suture the residual end of the cystic duct (arrow); E: The suture site of the residual end of the cystic duct (green arrow), the common hepatic duct (red arrow), and the CBD (black arrow); F: reinforce the suture site of the residual end of the cystic duct with absorbable clip (green arrow), the common hepatic duct (red arrow), and the CBD (black arrow). ACC: Acute calculous cholecystitis. CBD: Common bile duct. CBDS: Common bile duct stone. LC: Laparoscopic cholecystectomy. LTCBDE: laparoscopic transcystic CBD exploration.

**Fig. 3:**
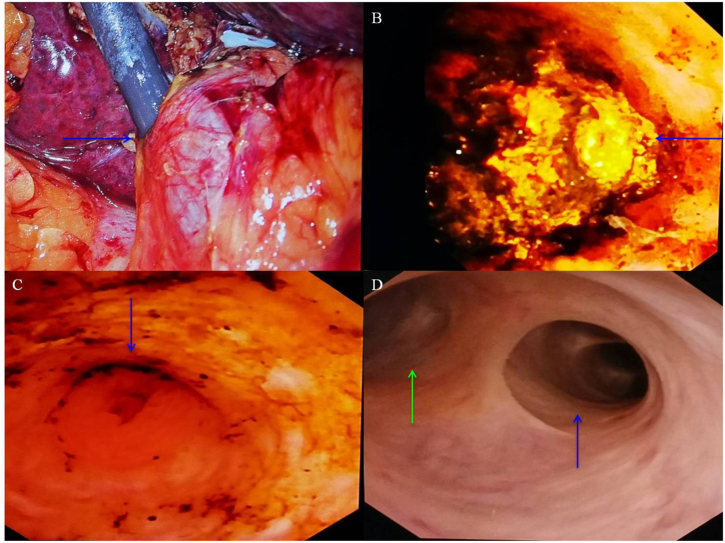
Intraoperative choledochoscopy images of LC + LTCBDE in the 96-year-old female patient with ACC + CBDS. A: The process of performing choledochoscopy through the cystic duct (arrow); B: A larger stone at the proximal end of the CBD (arrow); C: After completely removing the CBD stones, no stenosis was observed in the sphincter of Oddi (arrow); D: No stone was found in the left hepatic duct (blue arrow) and right hepatic duct (green arrow).

During the operation, the cystic duct was observed to have a relatively thin diameter (5 mm). The suture-needle puncture incision method was applied to create a horizontal incision in the anterior wall of the cystic duct at a distance of 1–2 mm away from the CBD (Fig. 2A). Following dilation of the Heister valve, a choledochoscope (Olympus CHF type 5, Japan) was inserted into the CBD via the cystic duct (Fig. 2, Fig. 3A). The larger stone was identified at the proximal end of the CBD (Fig. 3B). The crushed CBDS were removed through the choledochoscopic stone removal basket (Shanghai Innoway Medical Devices Co., Ltd. China, Fig. 2C). Following the removal of the CBDS, the suture site of the residual end of the cystic duct was reinforced with an absorbable clip after CBDS removal (Fig. 2D–F). Following the complete removal of the CBD stones, neither stenosis was observed in the sphincter of Oddi (Fig. 3C), nor was the residual stone found in the left hepatic duct and right hepatic duct (Fig. 3D). The operation lasted 220 minutes with an intraoperative blood loss of 100 mL. At the end of the operation, the patient was transferred to the general surgery ward. Following three hours of assisted ventilation, she was successfully weaned from the ventilator and the tracheal intubation was removed. Following postoperative cardiopulmonary function maintenance for 24 hours, the patient commenced a regimen of Bemiparin calcium (0.4 mL, subcutaneous injection, once daily), and gradually resumed her diet.

### 2.9 Pathological diagnosis

Acute cholecystitis, gallstones.

### 2.10 Outcome

On the 7th day following the surgical procedure, the patient exhibited no signs of discomfort, including abdominal pain or fever. A comprehensive review of cardiac function, chest CT, liver function, and blood routine, C-reactive protein, procalcitonin and other examination indicators revealed no abnormalities. The patient demonstrated a successful recovery and was discharged.

### 2.11 Follow-up

So far, the patient has been under close observation for a period of five months, during which time no abnormalities have been identified.

### 2.12 Final diagnosis

Acute calculous cholecystitis (ACC), common bile duct stones (CBDS).

## 3 Discussion

Currently, the global population is undergoing an accelerated aging process. It is estimated that by 2050, the proportion of the world's population over 60 years of age will nearly double, reaching approximately 2 billion older individuals. The latest data from the National Bureau of Statistics of China, released on January 18, 2024, indicate that the elderly population aged 60 years and above reached 296.97 million by the end of 2023, representing 21.1 % of the total population, indicating that China has entered a rapidly aging society.

Common bile duct stone is one of the most common diseases in world, which was found in approximately 10–15 % of patients with cholelithiasis. It occurs more frequently with advanced age. At present, the best treatment for choledocholithiasis is still controversial. The most widely accepted two techniques are endoscopicretrograde cholangiopancreatography (ERCP) plus laparoscopic cholecystectomy (LC) and laparoscopic common bile duct exploration (LCBDE) [8]. However, the safety and efectiveness of LCBDE in elderly patients with choledocholithiasis is still uncertain [2]. With the increasing popularity and technological advancements in laparoscopic and choledochoscopic procedures, LTCBDE has become a crucial minimally invasive surgical technique for the treatment of cholecystolithiasis complicated with choledocholithiasis complications [3]. Its primary advantage is the single-step resolution of gallstones and CBD stones without laparotomy CBD incision, thereby preserving the integrity and normal physiological function of the CBD. Additionally, LTCBDE is characterized by its minimally invasive, rapid recovery period, comprehensive stone removal, and pronounced therapeutic impact. Concurrently, this surgical procedure mitigates the risk of postoperative biliary stricture and stone recurrence caused by CBD suture, avoids bile leakage induced by CBD incision and prevents complications from T-tube drainage, thereby shortening hospital stay and recovery time [9]. However, the shortcomings of this surgical procedure are constrained by anatomical factors associated with cystic duct or larger CBD stones. The success of this procedure hinges on the expertise of the choledochoscopy operator, who must adhere to rigorous technical standards. The exploration of the branches of the hepatic duct is particularly complex, leading to significant variability in the application and success rate of this operation, as reported domestically and internationally [10]. Therefore, LTCBDE is not suitable for all patients with cholecystolithiasis combined with choledocholithiasis, and its success rate needs to be improved. It is essential to consider the aforementioned factors when selecting patients and refining surgical techniques to achieve better therapeutic outcomes [11,12].

Over the past four years, we have successfully treated 67 patients with cholecystocholedocholithiasis combined with acute obstructive suppurative cholangitis (AOSC), by means of LC plus LTCBDE on 28 patients, laparoscopic CBD exploration（LCBDE）on 37 patients, and laparoscopic transcystic approach with microincision of the cystic duct confluence in common bile duct exploration (LTM-CBDE) on 2 patients. Of these patients, 19 were over 80 years old and 5 patients were over 90 years old. It can be concluded that LTCBDE has significant advantages for elderly patients with AOSC, which provides hope for challenging the current mainstream status of endoscopic retrograde cholangiopancreatography (ERCP) treatment for elderly AOSC patients [13]. Our successful experience of LTCBDE shows that almost all secondary CBD stones or primary small (fine diameter ≤0.6 cm) CBD stones can be successfully removed with LTCBDE. Larger CBD stones may be removed after being crushed, by means of applying pressure and water flushing, pushing and crushing through the choledochoscopic stone removal basket. Key points for technological innovation and improvement include: carefully applying a suction device to fully dissect the neck of the gallbladder in the presence of prominent inflammation, edema, and adhesion; the complete dissociation of the cystic duct to its confluence, with a perpendicular alignment at approximately 90°; and reasonably applying the suture-needle puncture incision method to horizontally cut the anterior wall of the cystic duct 1–2 mm away from the CBD; accurately dilating the Heister valve of cystic duct; proficiently operating the choledochoscope; skilled stone-removal basket technique; if necessary, combined with holmium laser lithotripsy.

These innovations have the notable effect of markedly enhancing the success rate of LTCBDE. It is noteworthy that the advantages of applying the suture-needle puncture and incision of the cystic duct include: (1) Avoiding the reduction of the inner diameter of the cystic duct caused by tissue contracture when cutting the cystic duct with an electric hook or ultrasonic knife, which makes choledochoscope insertion difficult; (2) Overcoming the limitation of the cystic converging into the CBD at an acute angle downwards, facilitating the exploration of the CBD and the branches of the hepatic duct by choledochoscopy upwards; (3) Resolving the problem of Heister valve obstruction during choledochoscope insertion and improving the success rate of LTCBDE.

Although LTCBDE for elderly patients with cholecystocholedocholithiasis is considered safe and feasible, it is still rare for the super-elderly patients over the age of 90. We have reported [14] the clinical data of 372 elderly patients with biliary diseases, demonstrating that laparoscopic surgery in extremely elderly patients is safe and feasible, which key is to take measures such as actively treat preoperative coexisting diseases, strictly master operative indications, reasonably select surgical procedures, accurately carry out precise operation, strictly monitor and deal with intraoperative emergency, timely prevent and treat postoperative complications, especially focus on maintaining stable cardiac and pulmonary function during the perioperative period, and particularly strengthening the prevention and treatment of major adverse cardiac events (MACE) during the perioperative period [15].

This 96-year-old female patient with acute calculous cholecystitis and CBDS had a 20-year history of hypertension, coronary heart disease, and hyperlipidemia, as well as a 10-year history of right eye cataracts and glaucoma, resulting in blindness for 6 years; and a 10-month history of deep vein thrombosis in the lower limbs and pulmonary embolism. She had also been taking Rivaroxaban for an extended period prior to admission. Although ERCP treatment for elderly CBDS patients has a significantly superior therapeutic effect compared to traditional surgical procedures, which can notably reduce the treatment time of patients, enhance clinical efficiency, and effectively mitigate the incidence of postoperative complications. Preoperative endoscopic retrograde cholangiopancreatography plus laparoscopic cholecystectomy (pre-ERCP + LC) for the treatment of cholecystocholedocholithiasis has been demonstrated to result in a higher clearance rate of common bile duct stones, a lower incidence of postoperative bile leakage, and a higher incidence of pancreatitis compared to other approaches. Laparoscopic common bile duct exploration plus laparoscopic cholecystectomy (LCBDE + LC) may facilitate a shorter hospital stay [16]. Recently, reports have indicated that the preferred approach for treating CBDS is a laparoscopic-assisted transgastric ERCP after gastric bypass [17], which is feasible for CBDS after Roux-en-Y gastric bypass (RYGB) and can clear the duct primarily or following previous surgical exploration [18]. However, given that the patient's preoperative CT revealed a large common bile duct stone (diameter 15 mm), if ERCP treatment is to be employed, incision of the Oddi sphincter incision of, which increases the risk of postoperative reflux cholangitis, and the possibility of unsuccessful ERCP stone removal. Furthermore, ERCP treatment for elderly CBDS patients, who frequently exhibit compromised cardiopulmonary function and multiple underlying diseases, with the hallmark of multiple and large CBDS, can be challenging to tolerate prone position operations over extended periods. This restricts the applicability and operational duration of various ERCP techniques, ultimately leading to the inability to remove some stones. It is encouraging to note thatthis case was successfully treated by means of LTCBDE, utilising skilled laparoscopic and choledochoscopic techniques in conjunction with robust perioperative safety measures. The therapeutic outcomes achieved were satisfactory.

According to the scientific and technological novelty retrieval reported by Capital Medical University of China, there have been no other reports of LTCBDE for a CBDS patient over 96 years in the literature retrieved both domestically and internationally. This indicates that this case created a historical record for the highest age of 96 years for a super-elderly patient with choledocholithiasis to undergo successful treatment with LTCBDE both domestically and internationally.

## 4 Conclusion

In summary, this case illustrates that despite the high prevalence of preoperative comorbidities, elevated surgical risks, and a multitude of postoperative complications commonly observed in super-elderly patients with choledocholithiasis, LTCBDE remains safe and feasible. The principal factors are skilled laparoscopic and choledochoscopic techniques, adequate perioperative safety assurance, especially postoperative maintenance of cardiopulmonary function. This case provides an important reference and guidance for improving the treatment outcomes in super-elderly patients with choledocholithiasis.

## CRediT authorship contribution statement

**Zongming Zhang:** Writing – review & editing, Writing – original draft, Supervision, Investigation, Funding acquisition, Data curation, Conceptualization. **Limin Liu:** Investigation, Data curation. **Chong Zhang:** Investigation, Data curation. **Zhuo Liu:** Investigation, Data curation. **Yue Zhao:** Investigation, Data curation. **Hui Qi:** Investigation, Data curation. **Haiyan Yang:** Investigation, Data curation. **Baijiang Wan:** Investigation, Data curation. **Mingwen Zhu:** Investigation, Data curation. **Hai Deng:** Investigation, Data curation. **Jinqiu Feng:** Investigation, Data curation. **Fucheng Liu:** Investigation, Data curation. **Zhentian Guo:** Investigation, Data curation. **Peijie Yao:** Investigation, Data curation.

## Ethics statement

The study was approved by the Ethics Committee of Beijing Electric Power Hospital, State Grid Corporation of China, Capital Medical University, with the approval number of 2023083120101.

Written informed consent was obtained from the individual(s) for the publication of any potentially identifiable images/data included in this article.

## Data availability statement

All original data are available upon reasonable request to the first author and the corresponding author.

## Funding

This study was funded by the 10.13039/501100009592Beijing Municipal Science & Technology Commission (No. Z171100000417056) and the Key Support Project of Guo Zhong Health Care of China General Technology Group (GZKJ-KJXX-QTHT-20230626).

## Declaration of competing interest

The authors declare that they have no known competing financial interests or personal relationships that could have appeared to influence the work reported in this paper.
